# Predictors of Anxiety, Depression, and Stress among Female University Nursing Students during the COVID-19 Pandemic: A Cross-Sectional Study in Saudi Arabia

**DOI:** 10.3390/jpm12111887

**Published:** 2022-11-10

**Authors:** Zainab Fatehi Albikawi

**Affiliations:** King Khalid University, Nursing College, Community and Psychiatric/Mental Health Nursing Department, Khamis Mushait 39746, Saudi Arabia; zalbikawi@kku.edu.sa; Tel.: +966-5-6112-8667

**Keywords:** anxiety, depression, stress, family support, family salary, university students, COVID-19

## Abstract

**Background:** Students at universities increasingly struggle with mental health issues such as anxiety, depression, and stress. The present prevalence of these diseases may arise in the event of a crisis such as the coronavirus disease 2019 (COVID-19) pandemic. **Aim:** To estimate the level of anxiety, depression, and stress in female university nursing students, and to identify predictors for students’ anxiety, depression, and stress during the COVID-19 pandemic. **Methods:** An online cross-sectional descriptive study was conducted using a convenient sample of 115 female university nursing students. The Depression Anxiety Stress Scale (DASS-21) questionnaire was used to assess symptoms of anxiety, depression, and stress. Multivariate linear regression was used to identify predictors of anxiety, depression, and stress. **Results:** Stress, anxiety, and depression had prevalence rates of 23.7%, 18.5%, and 34.6%, respectively. Significant anxiety predictors included family support, family salary, being diagnosed with chronic illness, and being exposed to patients with COVID-19. Significant correlations were found between family support, family salary, family history of mental illness, and fear of being infected with COVID-19 and depression in female university nursing students. Students’ levels of stress were predicted by family support. **Conclusion:** The level of anxiety, depression, and stress among female university nursing students was determined to be moderate. It is advised that university nursing students receive interventions that support their mental health.

## 1. Introduction

The World Health Organization (WHO) declared the coronavirus disease 2019 (COVID-19) pandemic a public health emergency of international concern toward the end of January 2020 due to the disease’s rapid global spread [1]. Countries implemented a number of measures to interrupt the chain of infection, including stay-at-home orders, school and university closures, local and international travel bans, and social isolation. The limitations and effects of the illness can have a substantial impact on mental health [2]. Significant guidelines have been established by the World Health Organization (WHO) to address mental health issues that may lead to suicide [3].

Despite the fact that poor mental health is linked to a worse quality of life, diminished productivity, and disability, mental health is a crucial component of wellbeing [4]. Depression and anxiety are examples of psychological suffering that are connected to major medical diseases [5]. Some people find the educational process to be particularly difficult, especially university students, who feel academic, personal, and social stress during their academic experiences [6,7]. Nursing is one of the world’s most stressful professions, thus transitioning to a nursing career is incredibly difficult [8]. Nursing students have unsettling occurrences while they are learning, which have an adverse effect on their professional, academic, and personal lives [9].

The three main causes of stress among nursing students, according to the literature, are personal circumstances, clinical practice challenges, and academic worries [10]. For nursing students, in particular, the clinical practice setting can be a stressful and anxious situation [11]. The World Health Organization (WHO) has listed depression and anxiety disorders as two of the six leading causes of nonfatal health loss, responsible for 7.5% and 3.4% of years worldwide spent living with disability, respectively [12].

Both anxiety and depression are emotional reactions that produce a similar set of symptoms, such as exhaustion, trouble sleeping, muscle tension, and irritability. While anxiety is continuous even in the absence of a stressor, stress is frequently brought on by an external source and can last for a brief period [13]. Significant weight loss or gain, loss of interest in activities of daily living, altered sleep patterns, loss of focus, lack of energy, feelings of guilt or worthlessness, and even persistent thoughts of death or suicide are some of the symptoms of depression [14].

Anxiety is an unconscious reaction to depressive tendencies, which may turn into severe fear that leads to panic. Moreover, anxious students are reported to suffer from learning difficulties and an inability to solve problems. The physical symptoms that are caused by psychological distress include dryness of the mouth, shivering of the hands and lips, frequent urination, and restless sleep [15]. Existing literature on mental health problems indicates that university students are anticipated to be prepared to face future demands, stressors, and increased responsibilities in their academic and social lives.

Currently, it is believed that mental health problems are a very serious public health issue that accounts for one-third of all disabilities worldwide [16]. In Saudi Arabia, the COVID-19 epidemic caused 38% of students to experience moderate to extremely severe stress, fewer than half of students to experience moderate to extremely severe anxiety, and half of students to experience moderate to extremely severe depression [17]. The COVID-19 pandemic presents a larger risk to people’s mental health, not just in Saudi Arabia, but in many other nations as well. According to some researchers, university students between the ages of 18 and 25 experienced higher levels of stress, anxiety, and depression than students between the ages of 26 and 60 during the first phase of the COVID-19 pandemic. As a result, students may feel even more stress as they get used to the new online learning environment [18].

Numerous studies have shown that undergraduate university students suffer from significant levels of stress, anxiety, and depression [19,20]. The lifetime prevalence of stress was found to be 24.5% among undergraduate students in a study by Suleyiman and Zewdu [21], while the prevalence of anxiety symptoms was found to be 47.1% in a study carried out in Turkey [20]. According to a systematic analysis of research examining the prevalence of depression among college students, 10–84% of undergraduate students experienced depression [22]. These numbers demonstrate that university students frequently suffer from mental illnesses.

Prior to the pandemic, Alahmadi [23] performed a systematic evaluation in Saudi Arabia that comprised 19 publications and revealed that the prevalence of anxiety among students ranged from 34.9% to 65%. Overall, female university students had higher anxiety than male students. The World Health Organization (WHO) has warned that the coronavirus disease 2019 (COVID-19) has increased anxiety, depression, and stress. There is already a global rise in anxiety and depressive disorders [24]. As countries have seen a high increase in mental health issues, including anxiety, sadness, stress, sleep disorders, and terror among their citizens, the ongoing COVID-19 pandemic is causing psychological and emotional pandemonium [25,26].

The effect of COVID-19 on the mental health of university students has been researched to date in a number of nations, including the United States, China, Australia, Saudi Arabia, Jordan, and Bangladesh. Quarantine, a lack of physical activity, ambiguity about the illness, a lack of knowledge, and fear of contracting COVID-19 were revealed to be risk factors for the mental health state of university students in Bangladesh [27]. During the COVID-19 pandemic in Jordan, female university students scored higher in depression, stress, and anxiety tests than male students [28].

Additionally, anxiety over the chance of contracting an infection has been linked to poor mental health among Chinese university students [29]. In the United States, 90% of students reported greater financial anxiety during the pandemic [30]. During the pandemic, stress and depression were substantially more prevalent in younger individuals in Canada [31]. Physical health, resilience, and emotional support were all positively correlated with students’ mental health, according to Australian university students who reported having poor overall mental health throughout the pandemic [32].

Al Bahhawi, Albasheer [33] discovered that female university students in Saudi Arabia had significantly higher mean ratings for stress, anxiety, and depression symptoms. Therefore, the present study aims to estimate the level of female university nursing students’ anxiety, depression, and stress, and to identify predictors of students’ anxiety, depression, and stress during the COVID-19 pandemic.

Objectives of the current study.

- To estimate the level of female university nursing students’ anxiety, depression, and stress.
- Identify predictors of students’ anxiety, depression, and stress among female university nursing students during the COVID-19 pandemic.

## 2. Materials and Methods

### 2.1. Design

An online, descriptive, and cross-sectional assessment of the prevalence of anxiety, depression, and stress (ADS) and related risk variables was conducted for the current study. A population of female university nursing students participated in the current study.

### 2.2. Sample and Study Population

A power analysis was conducted using G*Power 3. The sample size was calculated considering a medium effect size (Cohen’s), with a statistical power of 80%, a probability level of 0.05, and an estimated sample size of 115 participants [34]. It requires 115 participants to take part in the study.

A convenient sampling method was used to recruit 115 female university nursing students in Saudi Arabia. The study population was only female university nursing students in Saudi Arabia because it was found that compared to men, women reported higher levels of anxiety and fear during the COVID-19 pandemic [35]. Previous studies on infectious disease outbreaks suggest that women suffer more social and psychological difficulties from the outbreaks than men [36]. Moreover, women in Italy, Spain, Jordan, and Turkey reported greater anxiety, depression, and acute stress than men [28,37,38,39,40].

Regular female university nursing students in their first, second, third, or fourth year of study met the inclusion criteria. The following students were excluded from the study: (a) visiting students enrolled for one semester in the nursing college; (b) interns practicing in hospitals; and (c) those who had received a diagnosis of any psychiatric or mental health conditions (assessed using the sociodemographic questionnaire).

Since their educational experience was in a new environment that could be the source of their psychological suffering, visiting students who came to study in the college for only one semester were excluded. Because their practice in the clinical field may be connected to their psychological discomfort, internship students are also disqualified. Additionally, due to the possibility that their levels of anxiety, depression, and stress were related to their disease, students who had any psychiatric or mental health conditions were eliminated.

### 2.3. Data Collection

The link to the tools was posted along with a notice about the study. A cover letter that outlined the study’s objectives and provided assurances regarding participants’ confidentiality and anonymity was posted alongside the link to the study tools. Students who accepted to take part in the study clicked the link to complete the Google form that contained the questionnaire. The utilization of online resources enables more affordable and timely access to individuals who are located far away. Online data collecting was automated, which saved the researcher time by doing away with the necessity for manual data entry [41]. Google Forms is a free online tool for designing and developing web-based questionnaires [42]. Participants were specifically identified, which determined how much control the research exerted over the selection. For complete control, eligible participants were selected according to specific characteristics [43]. The researcher checked characteristics from an existing nursing college database or mailing list to capture the necessary eligibility data. Moreover, the researcher controlled who could respond by issuing passwords by e-mail.

The study questionnaire consisted of mainly two sections: a sociodemographic questionnaire including age, marital status, family salary, family support, level of education, diagnosis of chronic illness, family history of mental illness, fear of being infected with COVID-19, and being exposed to a patient with COVID-19 in the last 2 weeks. Those variables were chosen according to the results of previous studies that were relevant to the current study objectives.

Due to its public availability, the Depression Anxiety Stress Scale (DASS-21) was used to evaluate the mental health of female university nursing students. It is sensitive to adolescence and measures depression, anxiety, and stress in the same form. The form was validated, and the construct validity of the Arabic version was well established [44]. The DASS-21 is a self-reporting survey with 21 items that were created to measure and evaluate stress, anxiety, and depression. The 21 survey questions are made up of a trio of self-reported scales for measuring stress, anxiety, and depression. The seven factors are rated from 0 to 3 on a Likert scale (0: “Did not apply to me at all,” 1: “Applied to me to some degree or some of the time,” 2: “Applied to me to a considerable degree or a good part of the time,” and 3: “Applied to me very much or most of the time”).

To determine the final score, the results of each subscale were multiplied by two. The manual states that the subsequent ratings are then categorized as “normal, mild, moderate, severe, or extremely severe” [45]. The Cronbach’s alpha values for the DASS-21 are 0.91 for depression, 0.84 for anxiety, and 0.90 for stress, making it simple to use and reliable. Numerous research has supported the DASS-21’s reliability and validity among university students [46].

### 2.4. Ethical Considerations

The Institutional Review Board granted its ethical approval (IRB). The authorization is ECM#2021-3802. Female university nursing students were made aware that taking part in the study was completely voluntary and that they could withdraw at any point by not responding to any of the questions. Their privacy was protected and only an aggregate of the results would be shared.

### 2.5. Data Analysis

Data were cleaned up before being analyzed with SPSS version 20. Analyses using descriptive statistics were carried out. For categorical variables, values were expressed as ratios and percentages; for continuous variables, they were expressed as means and standard deviations (SD). To determine the degree to which participant demographic variables predict anxiety, depression, and stress among the participants, multivariate linear regression was used. A priori, the probability for statistical significance was determined.

To classify the stress, anxiety, and depression predictors, three models were created. The linearity assumption was evaluated using scatterplots, the multicollinearity was assessed using the variance inflation factor (VIF), for which the value was less than 10, and the normality was verified using the goodness of fit test (Kolmogorov–Smirnov test). According to the hypothesis of homoscedasticity, the variance of the error terms is consistent across the ranges of the independent variables. To determine whether there was a significant difference in means between the independent variables and the three dependent variables—anxiety, depression, and stress—with other explanatory factors, a bivariate analysis was carried out using an independent *t*-test.

## 3. Results

### 3.1. Sociodemographic Characteristics of the Study Participants

The current study involved 115 participants in total. The participants’ average age was 22.2 years (SD: 0.65). The bulk of the participants, 98 (85%), were between the ages of 18 and 23; 108 (94.3%) were unmarried; 71 (61.1%) did not get family support; and 80 (69.2%) had a family salary of more than 8000 SAR. Eleven participants, or 9.7%, had been exposed to a patient with COVID-19. Regarding clinical features, the majority of participants (86.7%) were without a history of mental illness in their families, and 82.6% had no history of chronic illness. The fear of being infected with COVID-19 was experienced by more than half of the participants (54.3%) (Table 1).

### 3.2. Prevalence of ADS

Stress accounted for 34.6% of the participants’ psychological suffering. Additionally, 27 (23.7%) and 21 (18.5%) participants each exhibited anxiety and depression, respectively. Depression, anxiety, and stress all had mean DASS-21 values of 4.84, 5.43, and 12.46, respectively. Table 2 lists the prevalence of the three forms of psychological distress in order of severity.

### 3.3. Predictors of ADS

To determine whether there was a significant difference in the means between the independent variables and the ADS, Table 3 shows the bivariate analysis using an independent *t*-test. The findings showed that ADS was positively correlated with family salary, family support, having a chronic illness diagnosis, having a family history of mental illness, and fear of being infected with COVID-19. In the preliminary regression model, only factors with statistically significant relationships to the dependent variables were included. In order to construct the final linear regression model, the backward technique was used, and explanatory variables that were not significant were eliminated.

The final anxiety model’s parallelism to the constant was statistically significant (F = 24:3, *p* 0:001). Significant factors included family salary, family support, chronic illness diagnosis, and being exposed to a patient with COVID-19. R2 was 0.102 for the final model and 0.095 for the adjusted R2 (Table 4).

In the final model of depression, the effects of family salary, family support, family history of mental illness, and fear of being infected with COVID-19 were statistically significant compared to the constant (F = 17:189, *p* 0:001). In this model, R2 was 0.107 and adjusted R2 was 0.101 (Table 5).

In comparison to the constant, the stress final model had statistically significant differences (F = 24:3, *p* 0:001). Family support was an important factor. R2 was 0.132 for the final model and 0.116 for the adjusted R2 (Table 6).

## 4. Discussion

The frequency of anxiety, depression, and stress during the COVID-19 pandemic among female university nursing students was examined in this study, as well as any potential predictive factors. According to the findings of the current study, a striking number of female university nursing students had mild to severe anxiety, depression, and stress. In the current study, results for anxiety, depression, and stress were compared to those from general populations and university students both during and before the pandemic.

### 4.1. The Prevalence and Predictors of Anxiety

The current study found that there was a 23.7% prevalence of anxiety among female university nursing students during the COVID-19 pandemic, which is relatively consistent with the findings of other studies. For example, in China, the anxiety level was 22.6% [47]. Anxiety was prevalent (28.8%), according to a prior study carried out in the Philippines [48]. Additionally, Spain had a 21.6% anxiety rate [49].

However, the prevalence of anxiety in the current study was higher than that of university students’ anxiety during the pandemic in Saudi Arabia (7.3%) [50], and more than that in Italy (18.7%) [38]. Additionally, the current study’s prevalence of anxiety was lower than that of research done in Bangladesh (87.7%) [51], Jordan (67.9%) [28], and Japan (34.6%) [52]. The method employed to quantify anxiety, the sample size, and the existing sociocultural differences between the countries may all be possible causes for the observed variances.

According to the current study, students who were struggling financially showed more anxiety symptoms than other students. Likely based on Hamaideh, Al-Modallal [28] discovered a link between financial difficulties and poor mental health in university students [53]. The results of earlier studies are supported by the current study’s discovery that there is a negative link between anxiety and family support [49,54,55].

The presence of family support is thought to be a key component of psychological adjustment, which may help to reduce the pathogenic effects of stress [55,56]. Being close to family members is said to be a major factor in maintaining good mental health [28,57,58]. This outcome was consistent with earlier research indicating a link between anxiety levels and a lack of family support [59].

Students with chronic illnesses exhibited greater levels of anxiety, which is consistent with the findings of a study by Abdallah and Gabr [60] who discovered that university students with chronic physical conditions have been documented to experience psychological disorders such as anxiety. Being exposed to patients with COVID-19 in the previous week was found to be a predictor of anxiety in the current study, which is consistent with research carried out in the United Arab Emirates by Saddik, Hussein [61] who found that university medical students who interacted with COVID-19 patients experienced significantly higher levels of anxiety.

### 4.2. The Prevalence and Predictors of Depression

The current study found that during the pandemic, depression affected 18.5% of participants. This outcome was close to that of Japanese research on university nursing students, which revealed an 18.3% prevalence of depression [52]. However, the results of the current study are different from those of studies conducted in Jordan (78%) [28], Cyprus (57.25) [62], China (56.8%) [63], and Nagpur (96%) [64]. The differences in sample sizes and data-gathering methods may be the cause of these differences.

Based on the study’s findings, depression is significantly predicted by a family history of mental illness. This is consistent with research from Germany, India, and New Zealand that found people with family members who have mental illnesses are more likely to experience depression themselves [65,66,67].

According to the current study’s findings, which are in line with those of prior studies, depression is linked to poor family salary and a lack of support from family members [28,68,69,70,71]. In the current investigation, depression was found to be predicted by a fear of being diagnosed with COVID-19. Similarly, multiple studies revealed a link between depression and a fear of COVID-19 [72,73]. This fear has been noted in a study as a factor that influences depression [74]. Depression and COVID-19-dread have both been connected to higher rates of suicide in the general population [3].

### 4.3. The Prevalence and Predictors of Stress

In the current study, 34.6% of female university nursing students reported experiencing stress. Likewise, another investigation was carried out in Spain [75]. However, some researchers have indicated that stress is more common [28,76,77,78,79]. The recent findings clearly show that stress among nursing university students might be attributed to a lack of family support. Similar to Hamaideh, Al-Modallal [28], who found that being close to family members was a key stress factor, the current study discovered that nursing students who lacked family support experienced substantial stress. Similarly, a study conducted by Abdul [80] prior to the pandemic discovered that academic stress was prevalent among nursing students and was caused by family conflict 42.3% of the time.

### 4.4. Limitations

The present study has some limitations that should be considered when evaluating the results. Firstly, because the study was cross-sectional in nature, no causal processes connecting to the variables under examination can be found. This disadvantage may be overcome with a longitudinal study, but it would be time and financially-consuming. Future research is required to examine the long-term effects of the COVID-19 pandemic on the mental health of Saudi Arabian students across a range of educational levels and provinces. Furthermore, the sample was restricted to female individuals, which limits its generalizability.

Secondly, only participants who had internet access and who were sufficiently predisposed to be interested in the subject completed online surveys. The degree of bias in online questionnaires cannot be determined [81]. Therefore, it is advised that the research be repeated utilizing a paper survey approach and a bigger sample size. Thirdly, because the mental health of students was the subject of attention, the current analysis only looked at depression, anxiety, and stress; other common mental health issues were left out. The DASS-21 measure is not a diagnostic tool for stress, anxiety, or depression. Youths need to be subjected to more rigorous screening procedures for stress, anxiety, and depression, such as psychiatrist interviews.

### 4.5. Implications

Our understanding of how the COVID-19 pandemic affects female university students’ anxiety, depression, and stress may be improved by the study’s findings. Increased public and policymaker knowledge of the pandemic’s effects on mental health is necessary, and decision-makers at all levels should support citizen resilience. Some interventions, such as those based on resilience development and which are accessible online in Saudi Arabia, may be useful in promoting mental health outcomes among university students [82]. Some nations have begun offering virtual screening and consultation services for mental health [83].

According to the results of the current study, it is critical to assess the mental health of university students during the COVID-19 pandemic. Psychiatrists and mental health professionals are essential to reducing the likelihood of experiencing mental health issues during the COVID-19 pandemic [84]. Additionally, university students should be aware of the mental health help options available at their institutions. Governments must implement innovative strategies to divert the attention of young people, such as making government-made mobile phone calls in Oman free so that people can help and socialize with one another [85].

## 5. Conclusions

The current study compiles updated data that fills a known knowledge gap regarding mental health including anxiety, depression, and stress. The most recent research shows that female university nursing students are influenced by mental health issues. The current investigation showed that anxiety, depression, and stress are very prevalent. There was strong evidence that various sociodemographic traits and anxiety, depression, and stress were related. Governmental and academic institutions might use the study’s findings to create screening programs that are successful in promoting the mental health of university students. Concentrating on enhancing the mental health of students while they are engaged in academic work to improve the learning environment is urgently necessary.

## Figures and Tables

**Table 1:** Description of sociodemographic characteristics (*N* = 115).

Variable	Frequency	Percentage (%)
**Age (years)** 18–23 ≥24	98 17	85.0 15.0
**Marital status** Married Unmarried	7 108	5.8 94.3
**Family salary** <8000 SAR ≥8000 SAR	35 80	30.8 69.2
**Family support** No Yes	71 44	61.6 38.4
**Level of education** First year Second year Third year Fourth year	13 36 34 32	11.3 31.3 29.6 27.8
**Diagnosed chronic illness** Yes No	15 100	13.0 87.0
**Family history of mental illness** Yes No	20 95	17.4 82.6
**Fear of being infected with COVID-19** Yes No	62 53	54.3 45.7
**Exposed to a patient with COVID-19 in the last 2 weeks.** Yes No	11 104	9.7 90.3

**Table 2:** Prevalence of ADS (*N* = 115).

	Level	Frequency	Percentage (%)
**DASS-21** **Anxiety**	No anxiety	88	76.3
	Mild	9	8.2
	Moderate	10	8.7
	Severe	6	5.5
	Extremely severe	2	1.3
	Score: Mean ± SD		4.84 ± 5.73
**DASS-21** **Depression**	No depression	94	81.5
	Mild	8	7.3
	Moderate	5	4.6
	Severe	4	3.5
	Extremely severe	4	3.1
	Score: Mean ± SD		5.43 ± 7.08
**DASS-21** **Stress**	No stress	75	65.4
	Mild	13	11.4
	Moderate	19	16.7
	Severe	7	6.2
	Extremely severe	1	0.3
	Score: Mean ± SD		12.46 ± 8.06

**Table 3:** Predictors of ADS among nursing college students (*N* = 115).

Variables	Category	Anxiety M (SD)	*p*	Depression M (SD)		Stress M (SD)	*p*
**Family salary**	<8000 SAR. ≥8000 SAR	9.7(9.5) 7.4(7.6)	<0.001	17.0 (12.2) 12.1 (10.2)	<0.001	14.3 (10.3) 10.6 (9.6)	<0.001
**Family support**	Yes No	10.8(10.3) 7.2 (7.5)	0.054	20.0 (13.4) 12.7 (10.1)	<0.001	18.6 (12.6) 12.1 (10.3)	<0.001
**Diagnosed chronic illness**	Yes No	13 (10.2) 7.3 (7.5)	<0.001	17.6 (10.3) 12.8 (10.2)	0.005	19.2 (12.0) 13.2 (12.1)	<0.001
**Family history of mental illness**	Yes No	12.3 (7.5) 7.3 (7.6)	<0.001	17.5 (10.3) 11.1 (9.6)	0.002	20.8 (10.7) 12.3 (10.1)	0.002
**Fear of being infected with COVID-19**	Yes No	9.7 (8.3) 6.2 (6.5)	<0.001	22.0 (13.5) 12.7 (9.3)	0.005	19.1 (9.1) 12.1 (10.2)	<0.001
**Exposed to a patient with COVID-19**	Yes No	14 (10.1) 7.6 (7.9)	<0.001	21.2 (11.1) 12.7 (10.3)	<0.001	21.7 (10.6) 13.1 (10.1)	0.002

**Table 4:** Linear regression model of sociodemographic characteristics that predict anxiety.

Predictors of Anxiety	Unstandardized Coefficient B	Standardized Coefficient				95% CI for β	
		SE	Β	T	*p*	Lower Bound	Upper Bound
**Constant**	−10.785	2.745		−4.623	<0.001	−14.773	−6.786
**Family salary**	3.479	1.050	0.089	3.267	0.001	1.399	5.558
**Family support**	5.670	2.037	0.075	2.668	0.004	1.753	9.776
**Diagnosed chronic illness**	3.466	0.465	0.207	7.036	<0.001	2.229	4.202
**Exposed to a patient with COVID-19.**	5.490	2.036	0.068	2.744	0.004	1.653	9.668

**Table 5:** Linear regression model of sociodemographic characteristics that predict depression.

Predictors of Depression	Unstandardized Coefficient B	Standardized Coefficient				95% CI for β	
		SE	Β	T	*p*	Lower Bound	Upper Bound
**Constant**	−15.150	3.679		−4.616	<0.001	−15.150	3.659
**Family salary**	2.514	0.687	0.104	3.632	<0.001	0.768	2.541
**Family support**	5.457	1.653	0.106	3.472	0.001	5.457	1.653
**Family history of mental illness**	3.716	1.437	0.059	2.685	0.010	3.817	1.463
**Fear of being infected with COVID-19.**	5.87	2.764	0.056	2.100	0.028	5.778	2.567

**Table 6:** Linear regression model of sociodemographic characteristics that predict stress.

Predictors of Stress	Unstandardized Coefficient B	Standardized Coefficient				95% CI for β	
		SE	Β	T	*p*	Lower Bound	Upper Bound
**Constant**	−11.177	2.361		−4.822	<0.001	−16.035	−6.337
**Family support**	4.748	1.531	0.085	3.136	0.002	1.725	7.762

## Data Availability

The corresponding author will provide the datasets used in the current work upon reasonable request.
